# Supplementary material for: Skin Color Retention in Red Potatoes during Long-Term Storage with Edible Coatings
Source: Foods. 2021 Jul 2;10(7):1531. doi: 10.3390/foods10071531 (PMC8307985; doi:10.3390/foods10071531)
Supplement: Supplementary file 1 [file foods-10-01531-s001.zip › foods-1214951-supplementary.pdf]

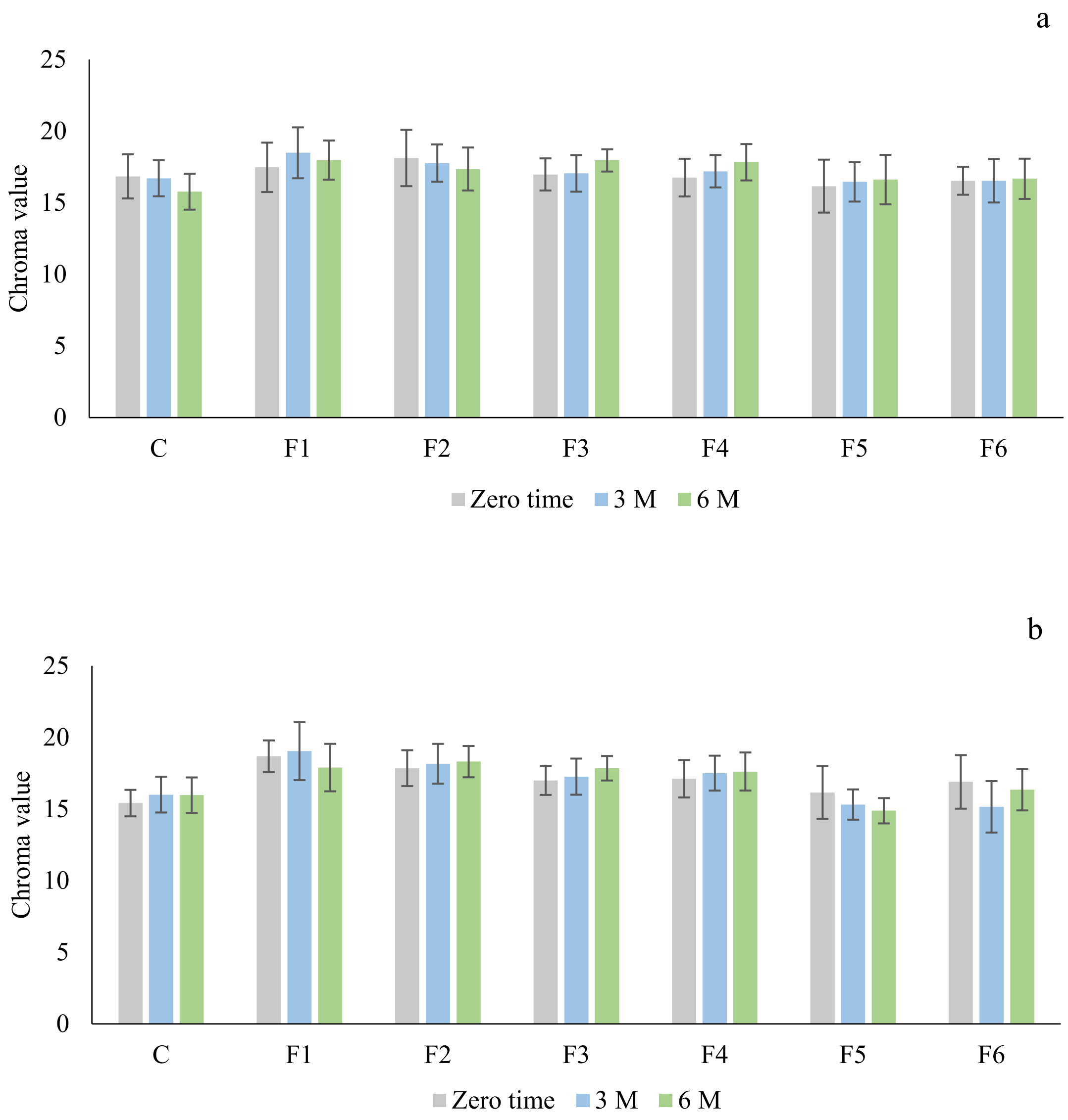

Figure. S1 a and b. Effect of coating on tuber chroma value of the Ciklamen (a) and Modoc (b) after six months of storage at 5 °C ± 1 and 90 % RH in the 2017 season. Data expressed as mean ± S.D., n = 3. C: control, F1: zein, F2: sodium alginate, F3: potato starch, F4:100 ppm methyl jasmonate, F5: chitosan, and F6 100 ppm Diphenylalanine

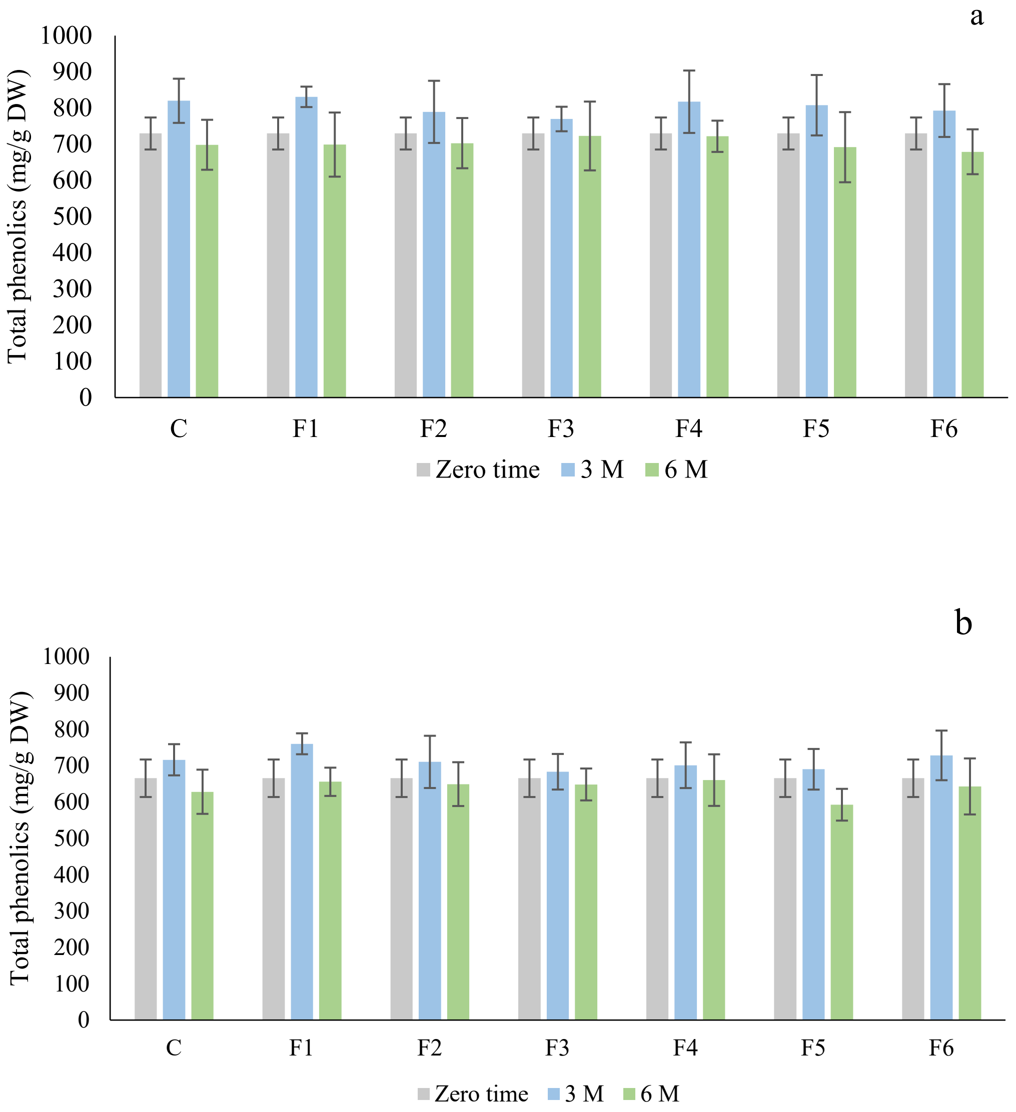

Figure. S2 a and b. Effect of coating on tuber total phenolics (mg/g DW) of the Ciklamen (a) and Modoc (b) after six months of storage at 5 °C ± 1 and 90 % RH in the 2017 season. Data expressed as mean ± S.D., n = 3 C: control, F1: zein, F2: sodium alginate, F3: potato starch, F4:100 ppm methyl jasmonate, F5: chitosan, and F6 100 ppm Diphenylalanine

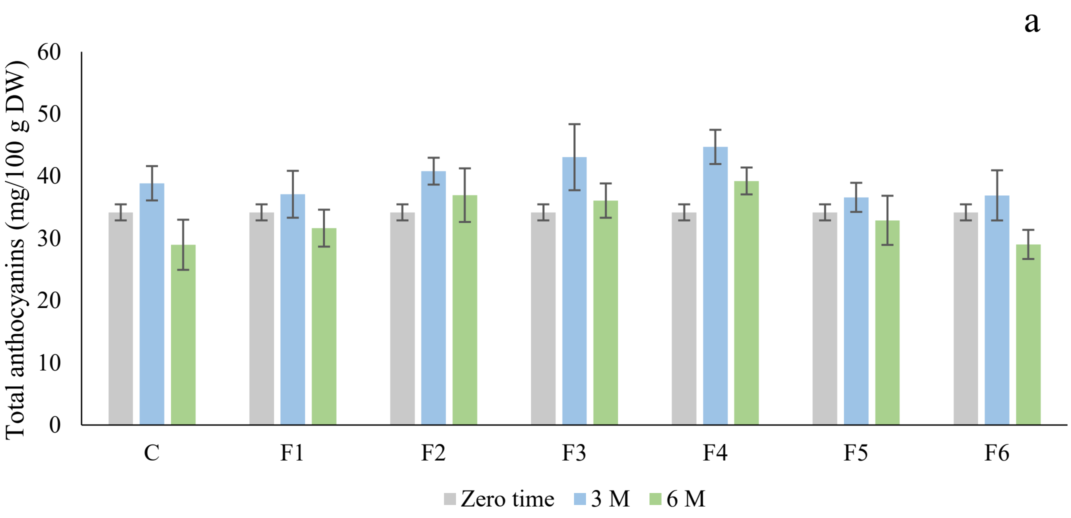

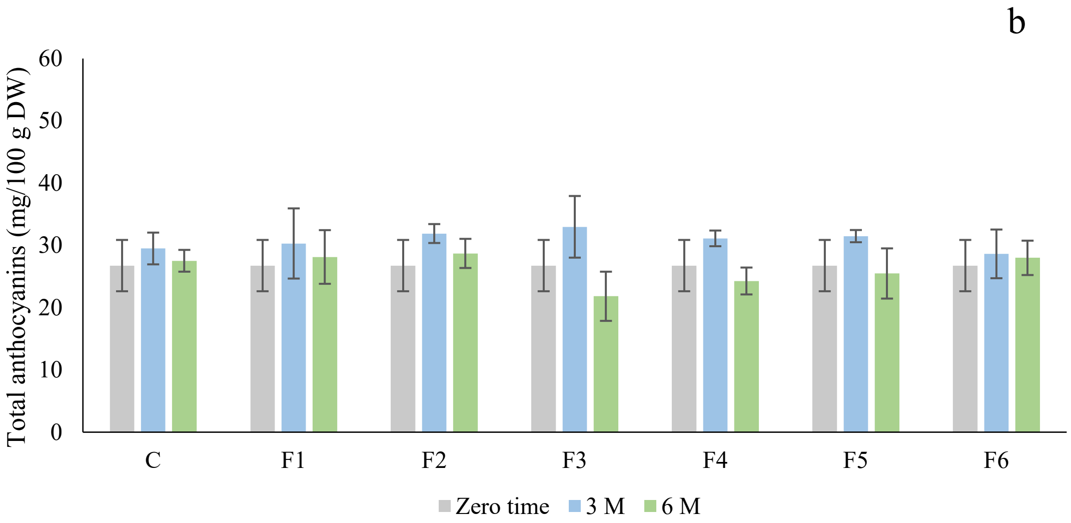

Figure. S3 a and b. Effect of coating on tuber total anthocyanins (mg/g DW) of the Ciklamen (a) and Modoc (b) after six months of storage at 5 °C ± 1 and 90 % RH in the 2017 season. Data expressed as mean ± S.D., n = 3 C: control, F1: zein, F2: sodium alginate, F3: potato starch, F4:100 ppm methyl jasmonate, F5: chitosan, and F6 100 ppm Diphenylalanine

a

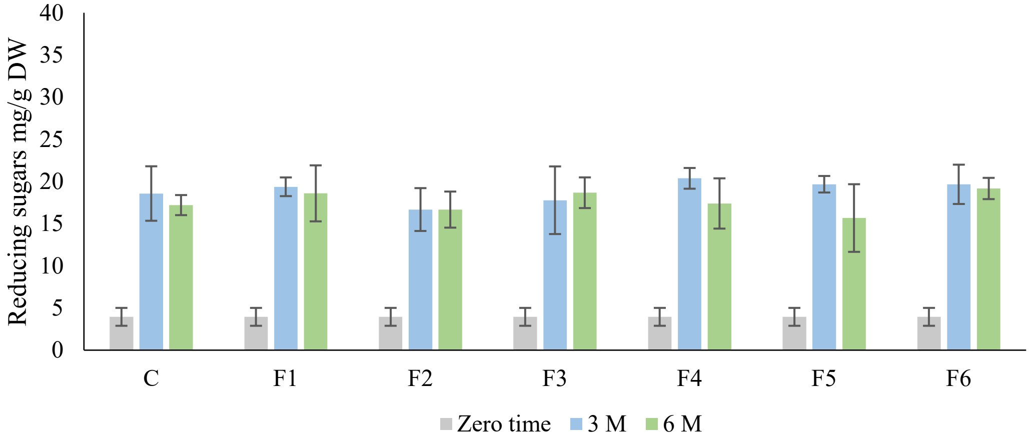

b

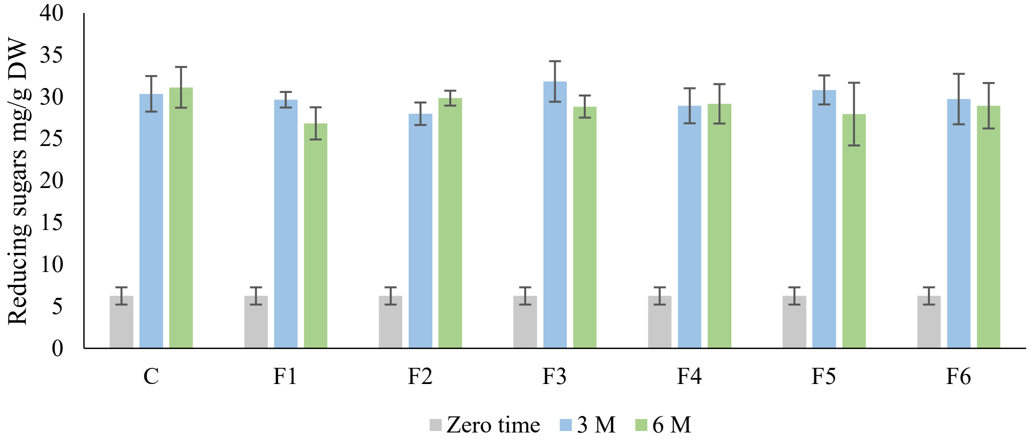

Figure. S4 a and b. Effect of coating on tuber reducing sugars (mg/g DW) of the Ciklamen (a) and Modoc (b) after six months of storage at 5 °C ± 1 and 90 % RH in the 2017 season. Data expressed as mean ± S.D., n = 3. C: control, F1: zein, F2: sodium alginate, F3: potato starch, F4:100 ppm methyl jasmonate, F5: chitosan, and F6 100 ppm Diphenylalanine

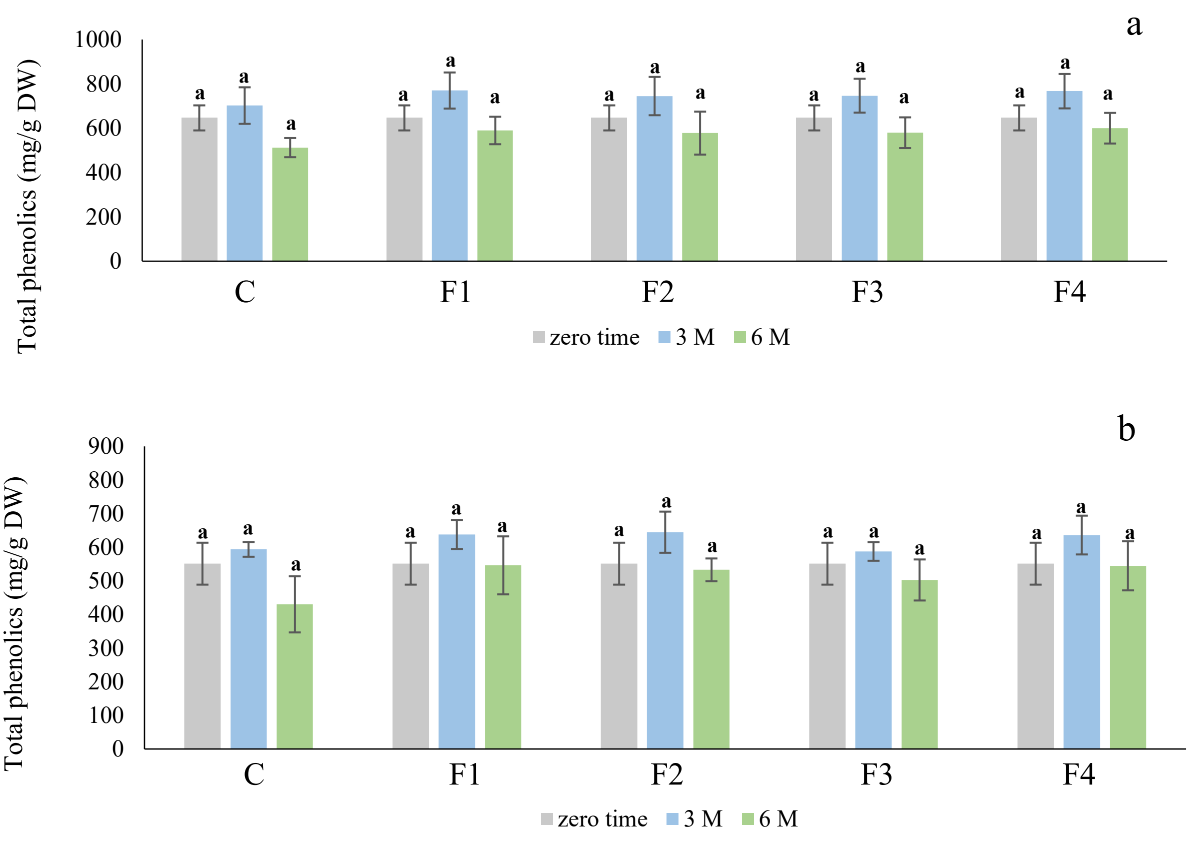

Figure. S5 a and b. Effect of coating on tuber total phenolics (mg/g DW) of the Ciklamen (a) and Modoc (b) after six months of storage at 5 °C ± 1 and 90 % RH in the 2018 season. Data expressed as mean ± S.D., n = 3. C: control, F1: sodium alginate, F2: potato starch + sodium alginate emulsion with oregano oil, F3: zein + chitosan emulsion with oregano oil, and F4: potato starch + chitosan + sodium alginate emulsion with oregano oil.

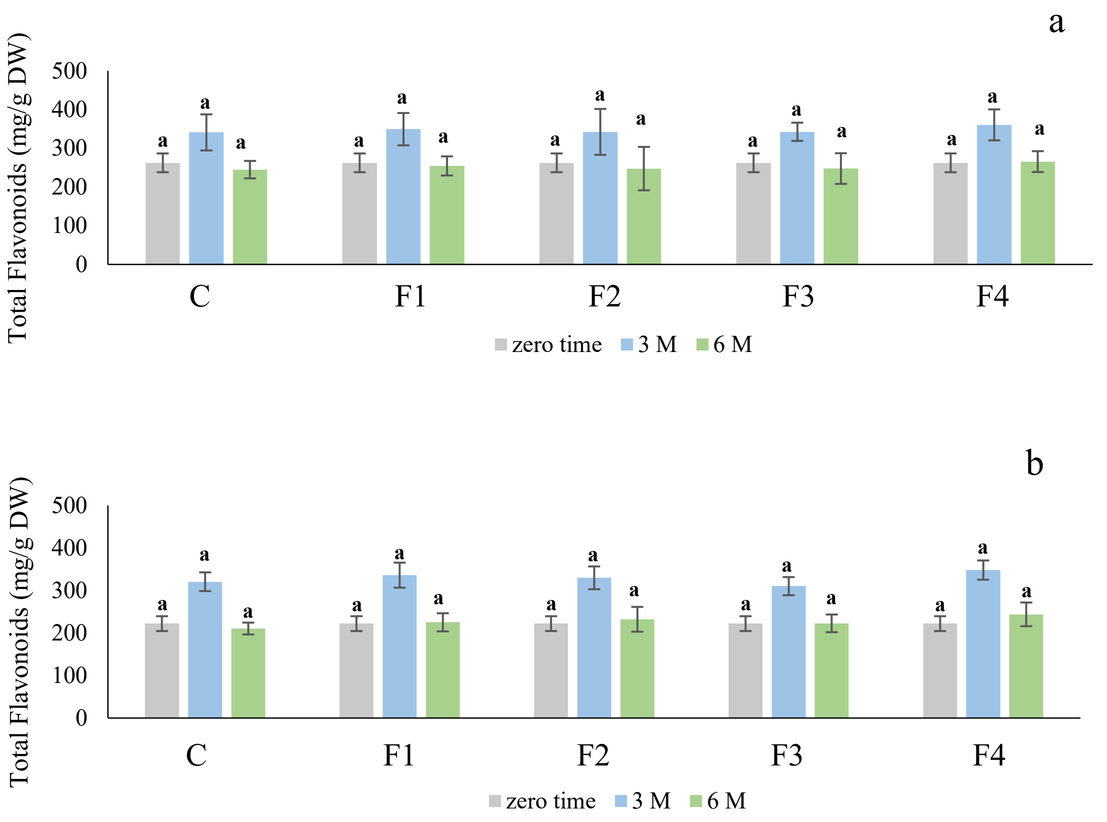

Figure. S6 a and b. Effect of coating on tuber total flavonoids (mg/g DW) of the Ciklamen (a) and Modoc (b) after six months of storage at 5 °C ± 1 and 90 % RH in the 2018 season. Data expressed as mean ± S.D., n = 3. C: control, F1: sodium alginate, F2: potato starch + sodium alginate emulsion with oregano oil, F3: zein + chitosan emulsion with oregano oil, and F4: potato starch + chitosan + sodium alginate emulsion with oregano oil.

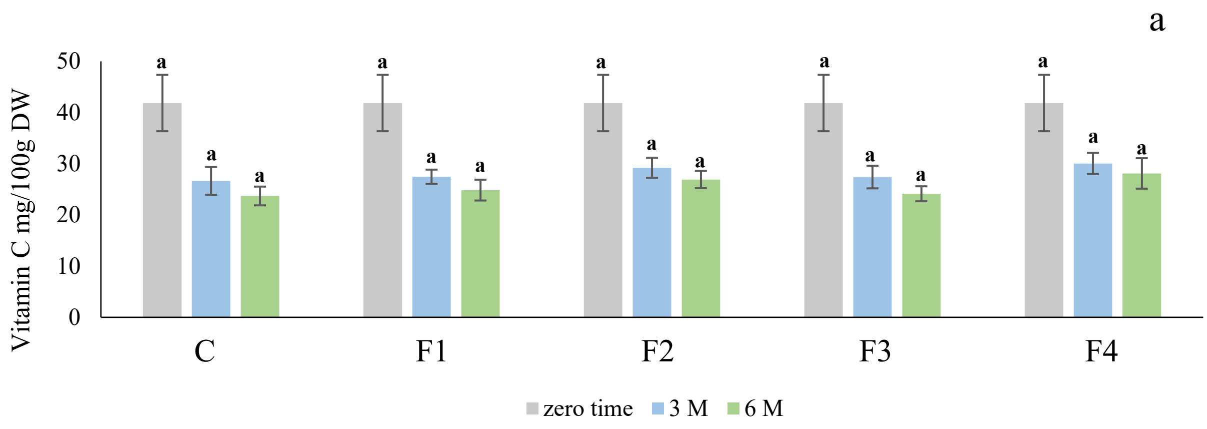

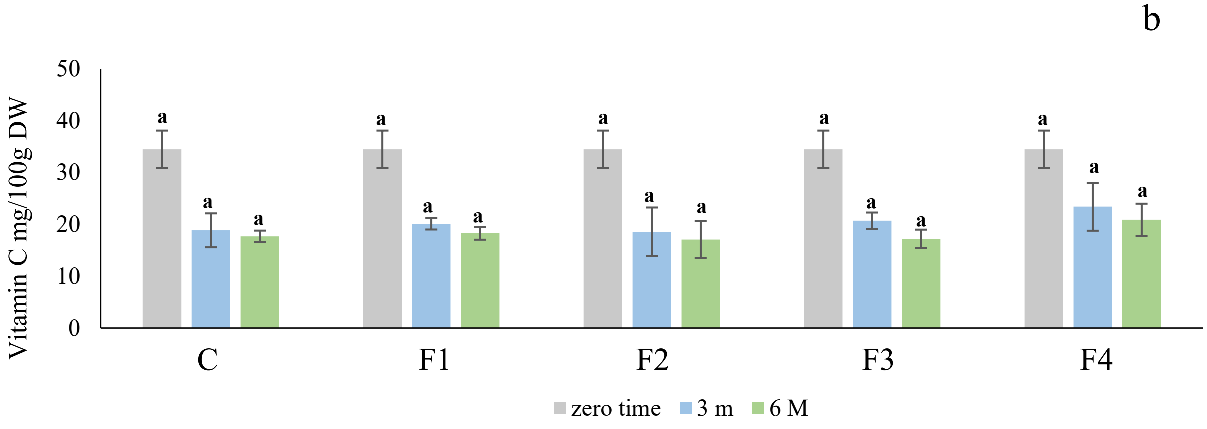

Figure. S7 a and b. Effect of coating on tuber vitamin C (mg/g DW) of the Ciklamen (a) and Modoc (b) after six months of storage at 5 °C ± 1 and 90 % RH in the 2018 season. Data expressed as mean ± S.D., n = 3. C: control, F1: sodium alginate, F2: potato starch + sodium alginate emulsion with oregano oil, F3: zein + chitosan emulsion with oregano oil, and F4: potato starch + chitosan + sodium alginate emulsion with oregano oil.

Table S1:

Analysis of Variance for color, anthocyanins, total phenolics, total flavonoids, reducing sugars, and Vit C. Table presents P values obtained using the ANOVA, General Linear Model function of R software, version 3.4.3.

Source	Color	Anthocyanin	Total phenolics	Total flavonoids	Reducing sugars	Vit C
Variates	0.571	0.237	0.062	0.483	0.000	0.000
Storage time	0.303	0.043	0.002	0.001	0.000	0.186
Treatments	0.000	0.030	0.856	0.992	0.132	0.476
Variates x Storage time	0.541	0.384	0.752	0.732	0.064	0.186
Variates x Treatments	0.000	0.486	0.956	0.992	0.012	0.686
Storage time x Treatments	0.000	0.536	0.992	0.903	0.967	0.997
Variates x Storage time x Treatments	0.000	0.048	0.993	0.907	0.970	0.989
